# Exploiting cancer genomics in pet animals to gain advantage for personalized medicine decisions

**DOI:** 10.1007/s13353-014-0206-0

**Published:** 2014-04-11

**Authors:** Magdalena Król, Tomasz Motyl

**Affiliations:** Department of Physiological Sciences, Faculty of Veterinary Medicine, Warsaw University of Life Sciences - WULS, Nowoursynowska 159, 02-776 Warsaw, Poland

## Introduction

Despite the first report of cancer being found in ancient Egypt, and since then an enormous knowledge improvement has been achieved, the patient’s prognosis is still unsatisfactory. Cancer is the second leading cause of death exceeded only by heart disease. According to the *United States Cancer Statistics Incidence and Mortality Web-based Report*, in 2014 every minute someone will die of cancer in the United States. Among malignancies, breast cancer is the most common tumour in the United States and Europe. Each year more than 1.3 million women worldwide are diagnosed with breast cancer and approximately 458,400 die from the disease (American Cancer Society 2011) despite the fact that breast cancer is highly curable if diagnosed and treated appropriately at an early stage. Annual breast cancer mortality has slightly decreased, however it is mainly related with an improvement of early diagnosis. Unfortunately in case of advanced tumours, medicine is powerless. The poor statistics and outcomes in mammary cancer patients make this tumour one of the most investigated in humans and domestic animals species.

In the bitch the incidence of mammary tumour is even three times over than in human (MacEwen 1990). About 50 % of all mammary tumours are malignant (Midsorp 2002). The incidence of mammary tumours is estimated at 31.8 and 20.4 in 100,000 uncastrated and castrated cats, respectively. Malignant neoplasms are found even more often in queens compared to neoplasms in bitches (MacEwen 1990; Midsorp 2002). Mammary tumours are very rare in other species. They have been reported sporadically in horses and only a few cases are known in ruminants and swine.

Epidemiology of mammary cancer in dogs and cats is similar to breast cancer. Humans and dogs have lived in similar environmental conditions for thousands of years; sometimes they even have a similar diet. Canine and human mammary tumours are usually of epithelial origin, they are also hormone-dependent. Moreover, in both species they occur spontaneously, in contrast to inducible or implantable mammary tumours in laboratory animals (Midsorp 2002).

## Comparative gene expression studies

One of the goals of the National Cancer Institute’s Comparative Oncology Program (NCI-COP) in the USA is to include companion animals with cancer in the mainstream of cancer research. It is easier to obtain samples from the dog than from laboratory animals (e.g. blood, urine, RTG). An important advantage of this model is the high similarity in the P450 cytochrome activity between dogs and humans. Location of P450 is not only limited to the liver, but they can be found also in other tissues, including cancer. Enhanced expression in tumour cells of these enzymes increases the potential for metabolism of anticancer drugs by the tumour cells directly. In chemotherapy, metabolism of drugs is performed at the site of the tumour, in a liver, or a combination of both (McFadyen et al. 2004). Thus, it is believed that results obtained in dogs can be more reliable for human medicine than results obtained in rodents. This is why the dog can constitute an intermediate stage between rodents and humans in clinical trials.

To follow aims of the American National Cancer Institute, Briggs et al. (2011) established a database of canine normal tissue gene expression data. It became possible after the public release of the canine genome project in 2005 (Lindblad-Toh et al. 2005). This genomic sequencing data, together with the development of canine-specific microarrays, provided the opportunity to conduct that kind of ‘omic’ studies in *Canis lupus familiaris*. Briggs and co-workers found tissue-specific gene expression profiles. Moreover, they have found similarities between gene expression patterns in canine and human healthy tissues. In both species, each organ could be clustered in a manner consistent with anatomical function and/or cellular composition (Briggs et al. 2011).

Several other studies cited below showed the same gene expression patterns and pathway alterations in human and animal cancers. These results confirm that dogs are good models to study spontaneous tumours in humans. An excellent study was conducted by Uva et al. (2009). The authors showed a close similarity between human and dog mammary tumours regarding the perturbation of many cancer-related gene sets and pathways, e.g.: PI3K/AKT, KRAS, WNT-beta catenin and MAPK, as well as a group of genes specific for cancer stem cells. Their data emphasized a high potential value of the dog as a preclinical model to test therapeutic agents because many of modern anticancer drugs target specific intracellular pathways. Importantly, their data also suggest the possibility of the reverse path, i.e. development of transcriptional biomarkers in dogs to be applied subsequently to humans.

Similarly, in both species: in dogs and humans, an up-regulation of the same ribosomal proteins in meningioma was detected (Thomson et al. 2005). This data show that canine brain tumours undergo similar genetic changes to those of human brain tumours on a molecular level. The similarity between both species seems to also be observed in lymphoma as in both species the co-expression clusters of NF-κB, p53, JAK/Stat and PI3K/AKT signalling pathways were found (Mudaliar et al. 2013). In addition, up-regulation of IL-10, IL-6 and IL-2 in canine lymphoma supported a role for inflammatory pathways maintaining the malignant phenotype (as observed in humans) and suggested that there may be common therapeutic targets in both species. Thus, canine lymphoma may represent a natural model of human disease and may offer a system to advance novel compounds to the clinic (Mudaliar et al. 2013).

Nowadays the possibility of full sequencing of cancer genome gives more research opportunities. An interesting study has been conducted by Navin et al. in 2011. They sequenced 100 single cells sorted from a heterogenous tumour and revealed four distinct groups of genomes. Sequencing is still a costly process, however technology is improving fast and demands are continuously decreased. International consortia perform large-scale analysis of cancer genomes that will provide in the future essential information on the landscape of cancer genomes (Vollan and Caldas 2011). Probably first reports of animal cancer genomes will be also published soon.

## Oncogenomics in diagnostics

**Fig. 1:**
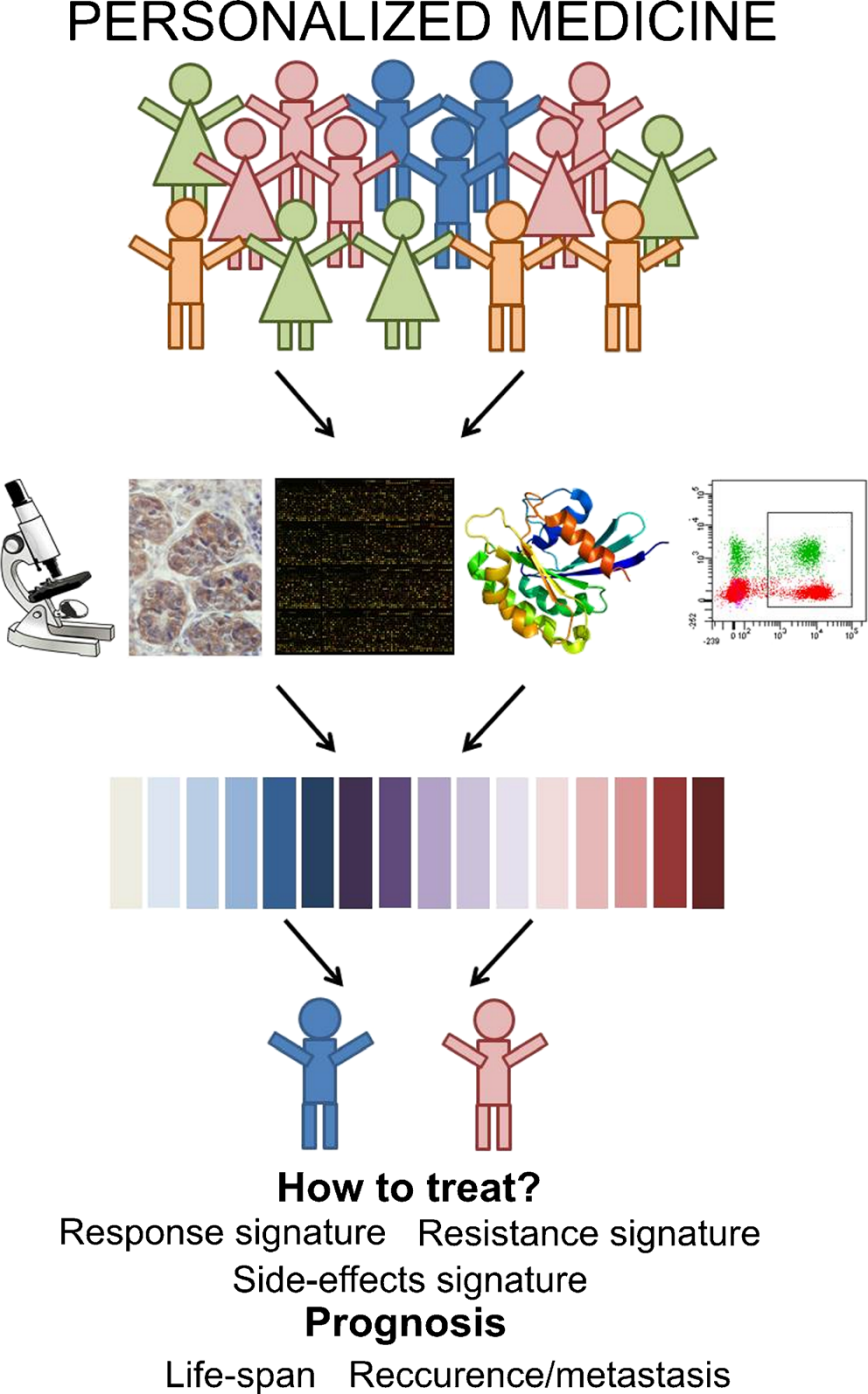
Personalized approach to medicine is based on the perturbations in a single cell genome or proteome and can help to decide which patients to treat and how they should be treated

Nowadays in all species challenges still prevail in the early diagnosis and management of cancer patients, due to unpredictability, in many cases, follow-up and response to adjuvant therapies. Despite wide availability of a variety of diagnostic methods (mammography, USG, RTG, CT, MR, PET), the microscopic examination still constitutes the most important one. Unfortunately, in most cases the value of histological prognostic indicators in predicting the course of a disease is weak. The histological diagnosis considers mainly the morphological properties of the tumours, however it does not account for the molecular mechanisms underlying cancer progression. For example, for some tumours the surgical removal of the primary tumour might be curative and the systemic therapy to eliminate any remaining tumour cells is not required. In other cases aggressive systemic chemotherapy is required after tumour resection. However, the pathological distinction between these cases is often unclear (van’t Veer and Bernards 2008). That is why investigators are nowadays focused on discovering novel predictive markers for different types of cancer. Such biomarkers are important to improve patient management. Scientists and physicians understood that the cancer therapy needs to change to a more personalized approach, in which each patient is treated according to the specific genetic defects in the tumour (van’t Veer and Bernards 2008) (Fig. 1). This breakthrough was possible due to the availability of large data sets about the gene expression in various tumours. An American group published a ‘milestone’ study in breast cancer describing four different molecular portraits of this disease representing the ‘tumour type’ itself, not only the particular tumour ‘sample’ (Perou et al. 2000). Based on the gene expression, the authors found four groups of tumours that might be related to different molecular features of mammary epithelial biology (ER+/luminal-like, basal-like, Erb-B2+ and normal breast). The most important point of this study was the clinical designation of two biologically distinct subtypes of tumours (which were previously classified as the same type), which should be treated as distinct diseases (Perou et al. 2000). Then, the 21-gene real-time rt-PCR-based assay has been patented as ‘Oncotype DX’ and used for tailoring the chemotherapy protocols in lymph-node-negative, ER-positive patients at early stage breast cancer (Hornberger et al. 2005).

The Dutch group went a step further and discovered a gene expression profile associated with the risk of early development of distant metastasis in young patients with lymph-node negative breast cancer (van’t Veer et al. 2002; van de Vijver et al. 2002). In this group of patients recurrence is likely in 20–30 % if surgery and localized radiation treatment are applied (van’t Veer et al. 2002; van’t Veer and Bernards 2008; van de Vijver et al. 2002). However, according to the European and American guidelines, about 85 % of women with this type of cancer undergo chemotherapy, mostly because conventional pathological parameters fail to reliably identify those patients who are likely to relapse. Therefore, 55–65 % of women with node-negative breast cancer are subjected to a toxic therapy from which they will not benefit but furthermore they will experience side effects (van’t Veer et al. 2002; van’t Veer and Bernards 2008; van de Vijver et al. 2002). Their microarray diagnostic test (patented as ‘MammaPrint’) based on 70-gene expression patterns was established as a powerful predictor of disease outcome in young breast cancer patients and to improve the guidance for the requirement of adjuvant therapy (van’t Veer et al. 2002; van’t Veer and Bernards 2008; van de Vijver et al. 2002; Glas et al. 2006) (Fig. 1). Personalized medicine can help to avoid unnecessary chemotherapy treatment and reduce the healthcare costs. However, at this moment the patient genotype information is not commonly included in treatment of breast cancer (Vizirionakis 2014).

In veterinary oncology personalized medicine is not used at all. However, some studies gave very promising results which bring hope for introducing modern diagnostic tests into the clinical settings in the future. For example, Monks et al. showed in 2013 that it is possible to provide a ‘personalized’ drug sensitivity and drug response signatures report of canine osteosarcoma to the veterinarian in five days from receipt of a sample. The group of Fowles have recently (2013) examined co-expression extrapolation (COXEN) method’s utility in a cross-species extrapolation of gene expression models (GEMs) to predict chemosensitivity for six anticancer drugs in canine osteosarcoma based on a human reference set. The GI50 ranges for all six drugs were comparable to the ranges calculated for the canine samples. COXEN analysis generated GEMs for each drug that on average were 79 % accurate in predicting sensitivity in the canine. Their analysis was also 73 % accurate in predicting disease free interval in canine osteosarcoma patients. Thus, they found that COXEN can be useful in cross-species prediction models and for future use in veterinary clinical trials.

The group of Dr Jan A. Mol from The Netherlands has designed a molecular-based method for discrimination of short and long survivors in canine patients with osteosarcoma. The authors showed that genes associated with cell cycle/proliferation, drug resistance and metastasis were commonly overexpressed in short survivors. Heat-shock proteins have been identified as involved in osteosarcoma development for both humans and dogs (Selvarajah et al. 2009). Comparison of the most important pathways for osteosarcoma development in humans and dogs revealed overlapping of seven of them, including Wnt, chemokine/cytokine, Alzheimer disease-presenilin pathway, fibroblast growth factor (FGF), platelet derived growth factor (PDGF), apoptosis and interleukin signaling pathways.

In the case of mammary cancer Klopfleisch et al. (2010) identified a gene expression profile of canine mammary tumours which was associated with an early metastatic spread to the lymph nodes. This differential expression profile contains several enriched functional gene classes and has 60 % overlap with expression profiles of metastatic human breast cancer described by the Dutch group (‘Mammaprint’). Metastatic tumours were characterized by increased expression of cell division genes and decreased expression of genes involved in focal adhesion. The dysregulation of cell cycle control is therefore a dominant feature of metastatic mammary tumours in various species.

In the case of canine mammary cancer, not only prediction of metastasis is important, but also the distinction between various grades of tumours. In veterinary medicine identification of ‘truly’ malignant tumours constitutes a real problem (Goldschmidt et al. 2011). The most significant criteria for the diagnosis of canine mammary malignancy are as follow: tumour type, nuclear and cellular pleomorphism, mitotic index, presence of randomly distributed areas of necrosis within the neoplasm, blood and lymphatic vessels invasion, and regional lymph node metastasis. Unfortunately, classification based on these criteria sometimes leads to the over-diagnosis of mammary carcinoma (Goldschmidt et al. 2011) especially due to significant human factors that may influence these results. As the highest grade of malignancy is associated with an increased risk of death within 2 years after mastectomy (Karayannopoulou et al. 2005), proper diagnosis may lead to prediction of the clinical outcome. Thus, our group performed gene expression analysis of canine mammary tumours of various grade of malignancy in order to find molecular markers of canine mammary malignancy (that could help to improve pathological diagnosis of the tumour grade). We have built the molecular classifier, which was able to distinguish the most malignant canine mammary tumours from the lowest estimated malignant tumours (Pawłowski et al. 2013a, b, c). Thus, based on the expression pattern of the *sehrl*, *zfp37*, *mipep*, *relaxin*, and *magi3* the pathologist could clearly distinguish grade III tumours which are correlated with poor prognosis. Optimization of this method is still in progress.

## Targeted therapy

Poor cancer statistics demonstrate the necessity of newer therapeutic modalities for achieving successful cancer treatment and cure. Knock-down of genes that contribute to cancer progression has been the goal of targeted genomics-based strategies, with the hope to selectively inhibit tumour growth with minimal side effects on normal cells. This has led to a recent acceleration in the development and optimization of tailored strategies for cancer therapy (Devi 2006). Targeted therapy has a great potential in oncology, but resistance to the agents is a significant clinical problem. For example, in colorectal cancer, it has been shown that treatment with Cetuximab (an EGFR inhibitor) is ineffective in the presence of an activating mutation of k-ras (Vollan and Caldas 2011). Another important mechanism of cancer cell self-protection is through the activity of multiple drug resistance transporters. This is frequently associated with over-expression of two or more membrane pumps which efflux anticancer drugs from the cytoplasm. These pumps protect tumour cells against the drug effects and correlated molecular processes. As increased efflux is such a significant contributor to a multidrug resistance in cancer cells, current research is aimed at blocking or inhibiting this specific mechanism. Because treatment of cancer cells with the use of classical inhibitors of efflux pumps often fails, we used a novel approach, that is: the specific RNAi to knock-down their expression (Pawłowski et al. 2013d). We showed that silencing of the efflux pumps had significant influence on cancer cell susceptibility to cytostatic drugs (determined based on the IC50 doses). Comparison of IC50 doses of anticancer drugs given in vitro and their maximum possible plasma concentrations showed that the *bcrp*, *mrp1* or *mrp3* knock-down can reverse cancer drug-resistance. Targeting of efflux pumps expression during chemotherapy could reduce the risk of anticancer drugs side effects by decreasing their doses. Our research is a completely new approach in the field of veterinary oncology. Despite the useful function of RNAi therapeutics for disease treatment, it still requires the development of clinically suitable, safe and effective drug delivery vehicles, there are some promising data from ongoing clinical trials that give hope for their practical application in the future (Pawłowski et al. 2013d).

A similar approach was used by Perez et al. (2011) who showed that inhibition of *MDR1* reversed inherent or acquired resistance to critical cytotoxic compounds for the treatment of cancer. Moreover, this approach allowed to lower chemotherapy doses maintaining tumour control. This is particularly relevant for paediatrics tumours where chemotherapy cures should be associated with minimum if no impact at all on long-term quality of life.

To sum up, there is a need of practical clinical utility of personalized medicine, especially in oncology (Vizirionakis 2014). Importantly as it has been recently proposed, by crossing the borderlines of genomics with nanotechnology and thus creating the necessary framework and infrastructure, the practical clinical utility of personalized medicine could be better served and advanced in a feasible and cost-affordable manner (Vizirianakis and Fatouros 2012). These efforts should be supported by the collaboration between companies, policy makers, research institutes, and hospitals (Yu et al. 2010). Nevertheless, proof will be required that ‘omics’-based personalized medicine generate an overall cost reduction before such an approach can finally fulfil the high expectations it has raised.
